# Classification of Breathing Signals According to Human Motions by Combining 1D Convolutional Neural Network and Embroidered Textile Sensor

**DOI:** 10.3390/s23125736

**Published:** 2023-06-20

**Authors:** Jiseon Kim, Jooyong Kim

**Affiliations:** 1 Department of Smart Wearables Engineering, Soongsil University, Seoul 06978, Republic of Korea; 2 Department of Material Science and Engineering, Soongsil University, Seoul 06978, Republic of Korea

**Keywords:** embroidered capacitance respiration sensor, dielectric, deep learning, breathing classification, conductive thread

## Abstract

Research on healthcare and body monitoring has increased in recent years, with respiratory data being one of the most important factors. Respiratory measurements can help prevent diseases and recognize movements. Therefore, in this study, we measured respiratory data using a capacitance-based sensor garment with conductive electrodes. To determine the most stable measurement frequency, we conducted experiments using a porous Eco-flex and selected 45 kHz as the most stable frequency. Next, we trained a 1D convolutional neural network (CNN) model, which is a type of deep learning model, to classify the respiratory data according to four movements (standing, walking, fast walking, and running) using one input. The final test accuracy for classification was >95%. Therefore, the sensor garment developed in this study can measure respiratory data for four movements and classify them using deep learning, making it a versatile wearable in the form of a textile. We expect that this method will advance in various healthcare fields.

## 1. Introduction

The ability to control various parts of life with healthcare equipment [1], body monitoring devices [2,3,4], safety instruments, planar waveguides [5], and other devices has been made possible by monitoring human activity. In particular, all living things on Earth undergo the fundamental physiological process of respiration. Humans breathe in and out in cycles to perform respiration. After breathing in oxygen-rich air, the respiratory system repeatedly exchanges gases across the alveolar–capillary membrane, such that oxygen enters the circulation and carbon dioxide leaves through the mouth and nose [6]. The representation of some respiratory parameters may serve as an indicator of a person’s physiological state [7,8,9] as well as mood and stress [10]. One effective method for observing and tracking human actions is the use of wearable technology [1,4], which combines e-textiles with wearable devices [11,12,13,14,15]. In addition, clothing capable of measuring respiration using an embroidery-type sensor [16] can be manufactured in the same manner as general clothing; therefore, it is a promising technology.

Generally, conductive fibers are used to create textile sensors. However, in this study, a unique approach was taken by using conductive threads to embroider a stretchable material. The use of threads instead of fibers has three main advantages. First, it provides flexibility, allowing the sensor to be embroidered in desired locations and enabling it to conform to the subject’s respiratory movements. Second, it offers comfort similar to regular clothing, allowing unrestricted movement and suitability for long-term wear, such as during sleep. Third, it maintains a nonintrusive nature, as the sensor is integrated into the fabric without the need for additional attachments directly on the subject’s body. These key properties enable accurate measurement of respiration.

However, there are some limitations associated with the embroidered textile sensor. Excessive sweating during vigorous physical activity may have a minor impact on the fabric-based sensor. Nevertheless, this can be addressed by using a film cover to protect the sensor [17].

**Figure 1:**
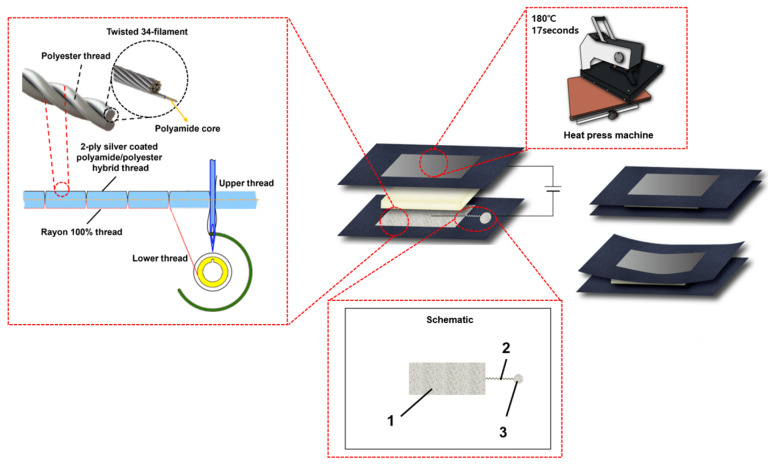
Mechanism for producing a capacitance respiratory sensor by embroidery method.

Breathing activity monitoring is a crucial aspect of effective computing approaches [18]. Body area networks (BANs), data processing centers, and distant assessment/cloud storage units are components of a typical wearable health monitoring system (WHMS) (Figure 1) [19,20,21,22]. Multiple on-body distributed biosensors, such as electrocardiogram (ECG), SaO2-oxygen saturation, and respiratory signal sensors, as well as inertial measurement unit (IMU) sensors, such as accelerometers, gyroscopes, and magnetometers, can gather various physiological and physical signals [23,24].

Speaker recognition [25], nonintrusive breathing monitoring [26], disease diagnosis [27,28], nonintrusive sleep apnea detection [29], diabetes screening, blood glucose monitoring, and respiration signals are regarded as crucial data [30]. Daily activities are linked to changes in respiratory patterns [31]. The study of respiratory parameters during different motions offers valuable reference data for treating diseases and managing health [32]. However, few studies have analyzed behavior by monitoring breathing patterns in real time. To solve this problem, real-time data classification is possible using sequence classification along with multiple data processing technologies, such as deep learning, which make it possible to implement medical and human activity recognition (HAR) by analyzing physiological and physical information.

In this study, we proposed a wearable integrated system that utilizes motion-based deep learning sequence classification for breathing categorization. The capacitance method was used to gauge the performance of the two electrodes (circuit and connector) made through embroidery with a conductive thread. The two electrodes were placed on the belly of a T-shirt, and the variable capacitance was measured in accordance with changes in the electrode distance and permittivity of the air in the lungs during respiration. This method uses a one-dimensional convolutional neural network (1D CNN) to measure, classify, and assess respiration. The 1D CNN used as part of the proposed system takes as input the respiratory signals measured from the embroidered textile sensor. The respiratory signals consist of sampled data over time, where each sample represents the signal value during a specific period of respiratory activity. These data are used as the input for the 1D CNN architecture.

The 1D CNN architecture comprises convolutional layers, pooling layers, and fully connected layers. The convolutional layers employ various filters to perform convolution operations on the respiratory signals, extracting temporal features. The pooling layers reduce the spatial dimensions and emphasize the extracted features. Finally, the fully connected layers use the extracted features as input for the final classification. For the classification task, various categories were used to classify respiration based on different activities. These activities included standing, walking, power walking, and running. The goal was to accurately classify the respiratory signals corresponding to these four activities using the algorithm.

In terms of evaluation, the accuracy of the validation data was considered to identify the point where the accuracy was highest without overfitting, as it was not higher than the accuracy of the test data. This allowed for determining the optimal results. Finally, research has shown that the proposed sensor has a wide range of design and structural applications in next-generation wearable electronic devices.

## 2. Experimental Method

### 2.1. Fabrication of the Embroidered Capacitance Respiratory Sensor 

Electrodes (circuits and connectors) were embroidered using silver-coated yarn (AMANN Silver-Tech, Houston, TX, USA) and an embroidery machine (Brother PR670E, Cranleigh, UK). A single conductive yarn was produced by twisting 34 filaments with silver-coated outer nylon cores. Upper and lower threads are the two types of threads used in embroidery. A nonconductive polyurethane (PU) film was placed on the exterior side of the clothing to protect the sensor, with the inner side acting as a conductor. The PU film attachment was carried out under the condition of 180 °C for 17 s using a heat press machine (INNOSTA ISP-450MR, Hanam-si, Gyeonggi-do, Republic of Korea). The electrodes were fabricated with a high density of 6 lines/mm. The connectors were constructed with medium density (4.5 lines/mm), which is the software’s default configuration because they are utilized for device measurements at the electrodes. We created a zigzag pattern (5 mm × 1 mm) that was supplied by an electric sewing machine to connect the line components linking the electrodes and connectors. The specifications of the sensors are listed in Table 1.

The respiratory sensors used in the experiment were fabricated with cloth. The selected cloth was the product with the best activity and elasticity (Sumnfit, Seoul, Republic of Korea), and it was made up of 87% polyester and 13% Spandex. The electrodes (1) were 100 × 50 mm in size, the connection line (2) linking the electrodes to the connector (3) was 335 mm in length, and the connector had a diameter of 20 mm. The electrodes were affixed to the abdomen. The connector piece that conveyed the output value was located close to the bottom of the electrode, which was chosen because it experienced the greatest change during respiration, to reduce any inconvenience when attached to the device while wearing garments. This mechanism is illustrated in Figure 1.

This silver-coated conductive yarn was specifically chosen for its excellent conductivity and low susceptibility to corrosion, making it an ideal choice for creating conductive surfaces [33], especially for applications such as sewing or embroidery [34]. Furthermore, as confirmed by the cytotoxicity test conducted in accordance with the Biological evaluation of medical devices standard (DIN EN ISO 10993-5), there was no cellular damage observed. Therefore, this yarn can be safely used in everyday life without causing harm to the human body [16]. The connection lines that connect the connectors and electrodes were designed using a zigzag pattern, which minimizes the impact on tension when wearing the garment-type sensor. 

**Figure 2:**
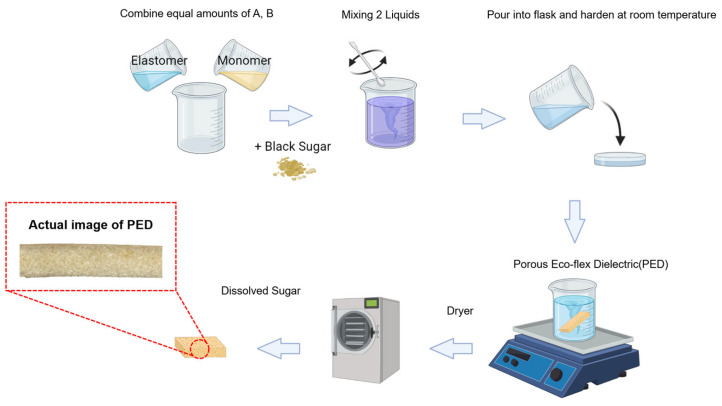
Porous Eco-flex dielectric (PED) production process.

Additionally, to replace the role of the lung before wearing the clothing-type sensor, Ecoflex (Smooth-on Inc., Macungie, PA, USA) was used for the experiments. The process of creating Eco-flex with pores and the finished appearance are shown in Figure 2. The production process was followed according to the product manual. First, substances called “Elastomer” and “Monomer” were mixed in a 1:1 ratio, and then brown sugar was dispersed in the solution. A 3D mold with dimensions of 100 mm × 50 mm was used to make the size identical to that of the electrode. The mixed solution was stirred at 120 rpm for 15 min to separate and evenly distribute the brown sugar powder. The mixture was cured at room temperature (30 °C) for 3 h. After the curing time, the 3D mold was used to remove the mixture, and the sugar was dissolved in boiling water using magnetic stirring at 200 rpm for a minimum of 24 h. The porous Eco-flex dielectric (PED) sample was obtained as a result of the presence of air pockets formed during the process.

**Figure 3:**
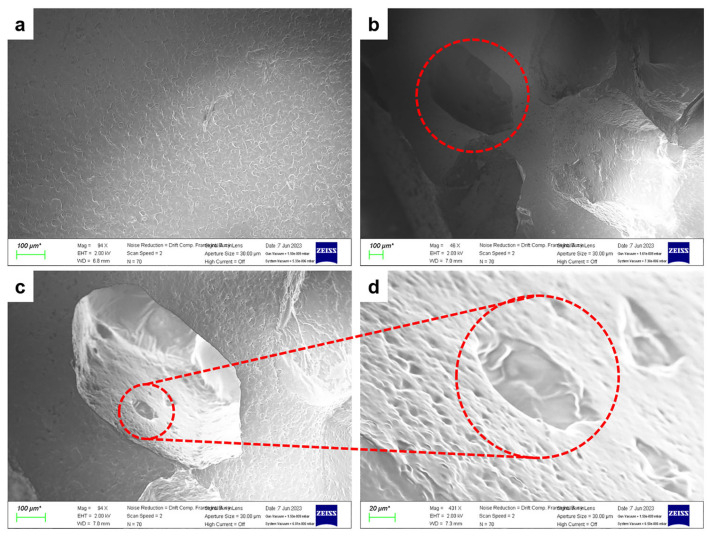
The morphologies of Porous Eco-flex dielectric (PED). (**a**) Before adding the sugar. (**b**) After dissolving in sugar, air pores formed. The presence of pores is clearly shown in (**c**,**d**).

The morphologies of the Porous Eco-flex dielectric (PED) before adding the sugar and after dissolving in sugar are illustrated in Figure 3. Figure 3a shows the scanning electron microscopy (SEM) image of a PED before the adding process with a smooth surface. Furthermore, the surface in Figure 3b is proof that the Eco-flex underwent uniform dissolution with sugar, resulting in the formation of air pores. The presence or absence of sugar is a crucial factor as it determines the presence of air pores, which can indicate changes in conductivity. Figure 3c,d demonstrate the structure of PED with air pores.

### 2.2. Respiration Data Collection Method

To collect respiration data accurately, the author went through the process. To select the appropriate frequency for respiration measurement, the author fabricated an Eco-flex that could serve as a dielectric in the experiment. The changes in capacitance between the two electrodes and the changes in capacitance due to the variation in dielectric properties of the air in the Eco-flex were measured. This helped determine the optimal frequency and the influence of dielectric properties.

Based on these results, a garment-type capacitance respiratory sensor was developed and used to measure respiration. When a person breathes, the volume of the abdomen changes, leading to a variation in the distance between the electrodes and resulting in changes in the measured capacitance. These changes are attributed to both the variation in capacitance due to the change in electrode distance and the change in dielectric properties of the air in the lungs. Therefore, the capacitance values, which vary due to these two factors during respiration, were measured and analyzed.

**Figure 4:**
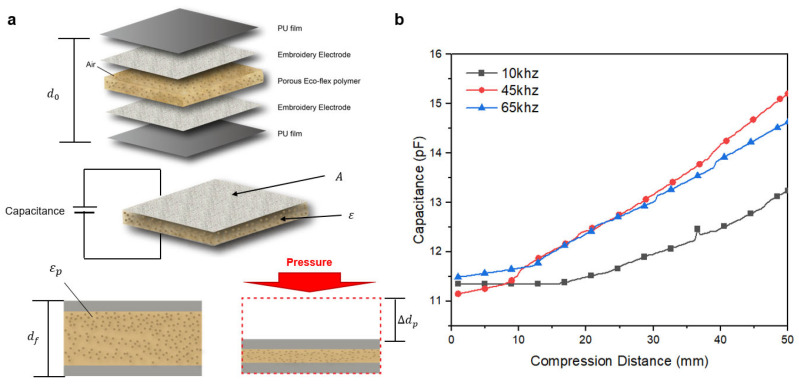

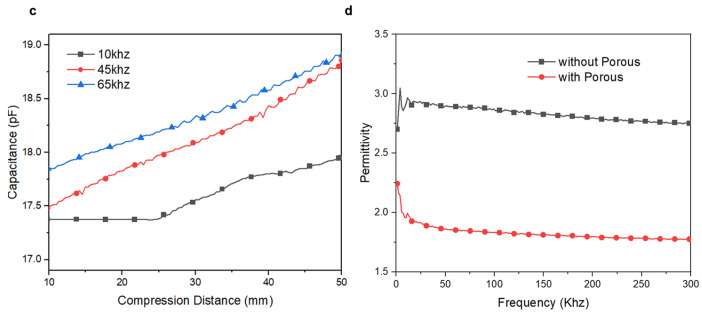
(**a**) Pressure-sensing mechanisms of the capacitive respiratory sensor using a porous Eco-flex dielectric (PED). (**b**) Capacitance changes according to compression distance (PED). (**c**) Capacitance change according to pressure by frequency (NPED). (**d**) Frequency dispersion of permittivity.

**Figure 5:**
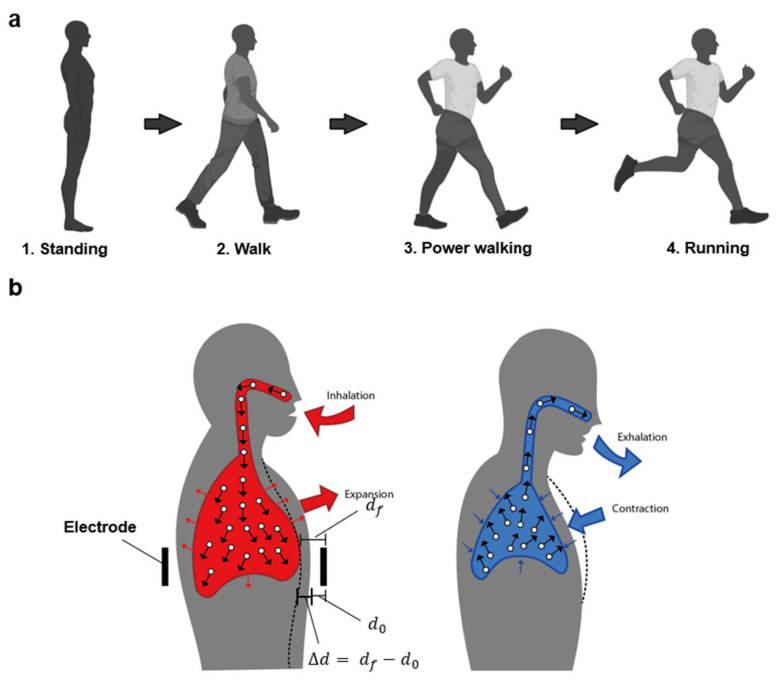
(**a**) Images of four actions (standing, walking, power walking, and running). (**b**) Changes in air volume in the lungs and abdomen induced by respiration.

As can be observed in Figure 4a and Figure 5b, the measurement method reveals that the distance between the electrodes changes with variations in air volume, dielectric properties of the lungs, respiration, and permittivity as a result of changes in air volume. Equation (1) explains this change in capacitance, where *A* is the electrode’s effective area, *ε_r_* is the material’s permittivity, *ε*_0_ is permittivity in a vacuum, and *d* is the thickness between the two electrodes.

(1) $$C=\frac{\varepsilon_{r}\varepsilon_{0}A}{d}$$

When an air layer is created using sugar in a porous Eco-flex (*ε_p_*), as shown in Figure 4a, the dielectric constant and distance between the electrodes (∆*d_p_*) change under pressure. Under pressure, the dielectric constant and distance between the electrodes (∆*d_p_*) change in the case of a porous Eco-flex (*ε_p_*), where sugar creates an air layer. Furthermore, the capacitance of the porous Eco-flex varies considerably, even under the same pressure, demonstrating that the shift in capacitance is significant [35]. When the pores are subjected to external pressure, the effective dielectric constant of porous Eco-flex (*ε_r_*), which may be explained by Equation (2) [36], rises [37]:

*ε_r_ = ε_air_ V_air_ + ε_Eco f lex_ V_Eco f lex_,* (2)

where *ε_air_* = 1 and *ε_Eco- flex_* = 2.8 [38]. *v_air_* stands for the volume fraction of air and *V_Ecoflex_* for the volume fraction of the impeccable Eco-flex. As the pores of the dielectric layer gradually closed owing to compression, the volume percent of air was reduced, and the volume fraction of Eco-flex increased. The effective dielectric constant of the porous Eco-flex composite increased as a result of the higher dielectric constant of the silicone elastomer (*ε_Eco-flex_* = 2.8), replacing the lower dielectric constant of the pores (*ε_air_* = 1) [35,39]. Because of the improved deformability of the porous elastomer, air gaps can be added to increase the sensitivity of the capacitance sensor.

To set up the basic measurements, we conducted an experiment by providing a difference in frequency between 10, 45, and 60 kHz using an LCR meter (E4980AL) for frequencies below 100 kHz. A pressure tester (Dacell Co., Seoul, Republic of Korea) was used to determine the difference in the distance between the two electrodes according to respiration. In addition, as a preliminary experiment to select the most suitable frequency for actual movements, we replaced the volume of air in the lungs with Eco-flex and measured the capacitance according to the change in the dielectric due to the change in air volume. We measured the capacitance according to the change in distance between electrodes due to pressure and the change in dielectric due to the change in air volume and selected the 45 kHz frequency, which showed the highest change rate (as shown in Figure 4b) [16]. Furthermore, when comparing the nonporous Eco-flex dielectric (NPED) using the same three frequencies, it also exhibited the largest amplitude of change at 45 kHz. Compared to PED, the amplitude difference of PED ranged from 11 pF to 15 pF, showing a difference of approximately 4 pF, while NPED ranged from 17.5 pF to 18.75 pF (Figure 4c), indicating a difference of 1.25 pF. Therefore, as the dielectric properties exhibited greater variation with air expulsion, resembling the actual changes in lung air volume, PED was chosen for the experiments. The variation in air volume is represented by the dielectric constant, as shown in Figure 4d, which presents the results measured at frequencies ranging from 1 kHz to 300 kHz. In this graph, the presence or absence of air pores can be observed through the difference in the dielectric constant.

To analyze the respiration according to movement, we wore clothing-type respiration sensors and performed four types of actions: standing, walking, power walking, and running. All actions were repeated five times for 1 min each, and the respiratory rate was typically 12–20 breaths/min. Standing was measured as remaining still in the attentional position without moving. Walking involved walking at a normal pace while swinging the arms lightly. Power walking was performed at a slightly faster speed than walking, with the arms swinging more vigorously. Running was performed at a speed 1.5 s faster than power walking, with more bending of the knees and greater movement of the upper and lower body (shown in Figure 5a).

As shown in Figure 5b, the capacitance value is measured by the change in the distance (*d_f_*) between the electrodes located at the front and back of the body, depending on the volume change of the body. Therefore, according to the principle of capacitance, the capacitance changes because of the momentary difference (*d_f_* − *d*_0_). Second, during this process, the dielectric constant of air changes owing to the change in the lung volume, which affects the capacitance value.

Breathing causes the amount of air in the lungs to increase, which lowers the dielectric permittivity. Under pressure, the dielectric constant of the skin layer increases [40], causing the capacitance to increase. The amount of air and the separation between the two electrodes are the other two elements, which have a greater impact than this one does. However, during exhalation, the abdomen contracts, moving the electrode below and raising the capacitance.

### 2.3. Classification of Respiration Data According to Motion

In this study, the difference in the capacitance of the data obtained from the respiration sensors throughout a time window corresponding to the four motions of interest was interpreted as the respiratory cycle. Compared to traditional methods, deep-learning-based solutions are more adaptable to complicated and abstract sets of data because they require fewer manual procedures [41]. In particular, 1D CNNs have received considerable attention because they are useful for classification and have a shallow architecture that is easier to train and run. With lower hardware setups and costs, they are suitable for various applications such as recognizing interesting patterns [42,43,44,45]. In this study, we used a 1D CNN to divide the four categories of standing, walking, power walking, and running from time-series data [46,47]. The breathing cycle and activation pattern were continually learned during the operation to provide the correct classification.

The electrodes were positioned at the upper abdomen (just above the umbilicus) and its corresponding posterior side, showing the largest amplitude of change during respiration. In the case of an average individual, the volume of the abdomen undergoes the most significant variation during breathing, and among various electrode placements; thus, this specific location was determined to be the most suitable for accurately measuring abdominal volume changes [17].

**Figure 6:**
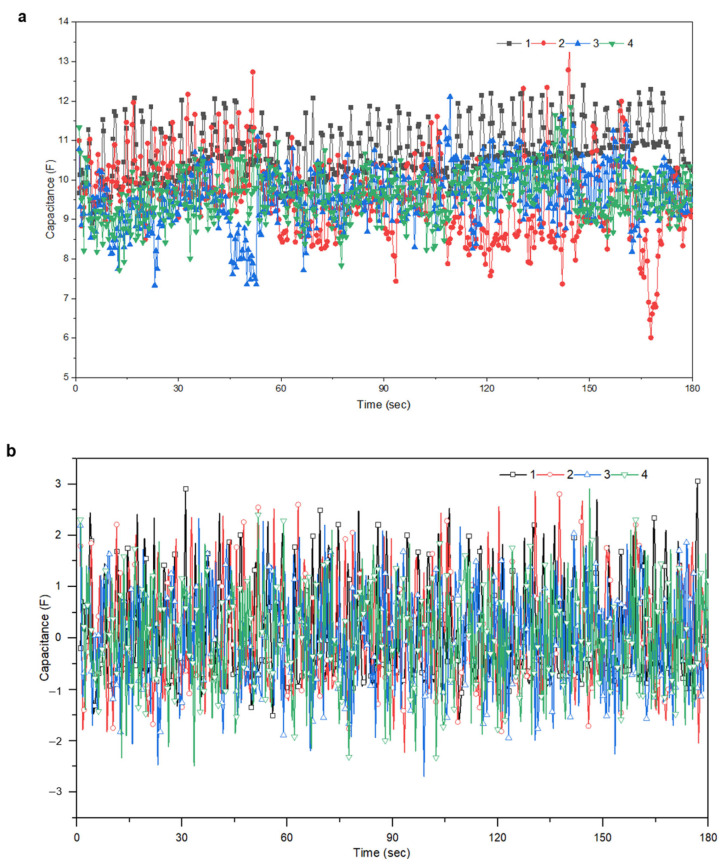
Breathing data according to movement (1. standing, 2. walking, 3. power walking, 4. running), (**a**) before normalization, (**b**) after normalization.

When inhaling, the abdomen expands and volume increases, whereas exhaling causes the abdomen to relax and volume to decrease. Additionally, volume changes differ depending on each movement, and changes in the respiratory cycle can also occur. The measured values for the four items have distinct lengths and patterns as well as variable scales of capacitance values because the respiratory cycle of the motion to be measured is altered and measured directly by the person wearing it. The complete dataset comprised 20 sets, each of which ended in a time series corresponding to a single channel. As shown in Figure 6, owing to the possibility of human error in performing four specific actions manually, the data were normalized to provide accurate training data. So, the normalized dataset retains the shape properties, including the same skewness and kurtosis as the original raw dataset and has a mean of 0.

(3) $$Z=\frac{X-\mu }{\sigma }$$

The calculation of the standard score (𝓩) for each raw score (𝓧) in a single time series within each dataset involved using the mean (*µ*) of all data points in the set and the set’s standard deviation (*σ*), as shown in Equation (3). 

**Figure 7:**
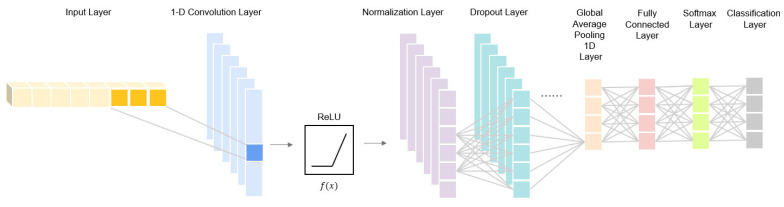
Architecture of the 1-D convolutional neural network model.

The 1D CNN model architecture (as shown in Figure 7) used in this study consists of several layers. The first layer after the input time series is a convolutional 1D layer with dimensions of 23 × 4 × 1. Each filter in this layer implements a convolution, and the resulting outputs are then passed through the rectified linear unit (ReLU) activation function. The ReLU function determines the neurons in the network that should be activated based on whether their input values are significant. If the input value is less than or equal to 0, the function returns a value of 0; otherwise, it returns the calculated value. This function is linear when the input values are positive and nonlinear when they are negative because it returns to zero in these cases. The activations of the previous layer are then normalized using a normalization layer, which transforms the activations to a standard deviation close to 1 [48] and a mean activation close to 0.

To prevent overfitting, a dropout layer was employed with a dropout probability of 0.5, which randomly deactivates certain neurons in the layer. This layer acts as a mask, nullifying or randomly setting some neuron inputs to 0, while scaling the remaining inputs to maintain the same total input [49]. The same series of convolutions was repeated one more time through the dropout layer.

The output of the 1D convolutional layer was then transformed into a 1D vector using a 1D global average pooling layer. A fully connected layer with an output size of four or more classes maps the output of the layer to a vector of various probabilities. The softmax layer follows, and finally, the classification layer. The softmax layer applies a normalized exponential function to the input, converting a vector *z* of *K* real values into a vector *K* of probabilities, where the sum is 1. The input values of the function, which can be positive, negative, or zero, are transformed into values between 0 and 1, allowing them to be represented as probabilities. The following Equation (4) defines a function when *K* is greater than 1:

(4) $$\sigma {\left(z\right)}_{i}=\frac{e^{z_{i}}}{\sum_{j=1}^{K}e^{z_{j}}}\;for\;i=1,\ldots ,K\;and\;z=\left(z_{1},z_{2},\ldots ,z_{K}\right)\epsilon R^{K}$$

In this study, we divided the overall respiratory data for each of the four actions (1–4) into 20 sets and fed them into cells. Each set contained one input received from the capacitance sensor and four respiratory outputs corresponding to the four actions. The data were categorized into three types: training, validation, and testing. A total of 157 datasets were used, with a training–validation–testing ratio of 70:15:15. MATLAB R2023a (Mathworks Inc., Natick, MA, USA) was used to train the 1D CNN, with a learning rate of 0.001 and a minibatch size of 6 for 1000 epochs.

## 3. Results

### 3.1. Capacitance Properties of the Embroidered Respiratory Sensor

**Figure 8:**
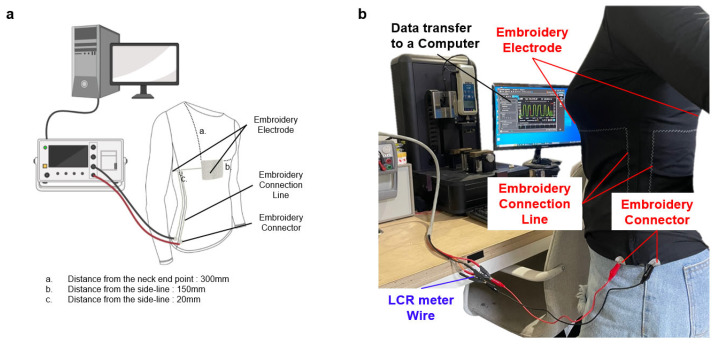
(**a**) Schematic of the universal testing machine. (**b**) Measurement of respiratory capacitance sensors in clothing.

Basic respiratory measurements were obtained using a garment-based respiration sensor at 45 kHz. The subject was seated in a relaxed position for 1 min while natural breathing was recorded. The sensor was designed with embroidery to seamlessly integrate with clothing, and a cable was used to connect it to an LCR meter for measurement, as illustrated in Figure 8. The distance from the electrode to the shoulder endpoint (a) was 300 mm, the distance from the electrode without the connecting line to the lateral line (b) was 150 mm, and the distance between the connecting and lateral lines was 20 mm.

**Figure 9:**
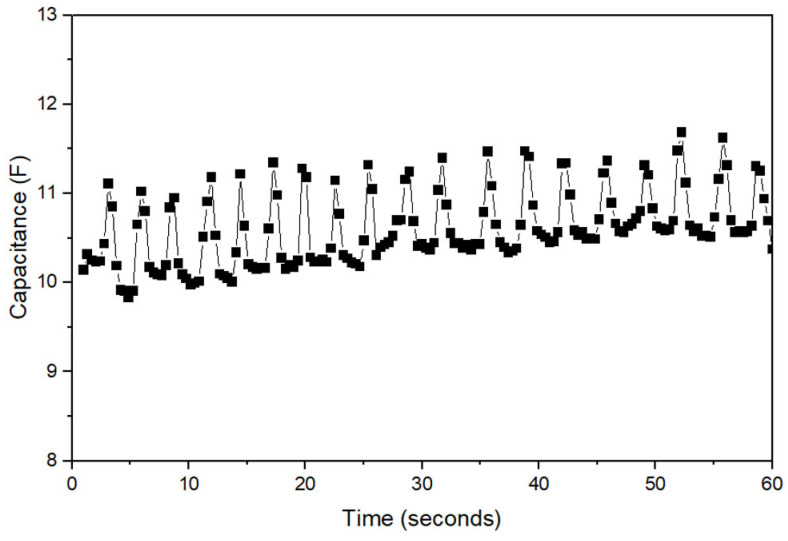
Normal respiration data.

Real-time data were transmitted and monitored on a PC during the measurements. As shown in Figure 9, 19 respiratory cycles were observed within 1 min, with measurements ranging from approximately 9.5 F to 12 F. Because the sensor was designed without volume or weight, it did not interfere with the data output and performed similarly to an IMU. During inhalation and exhalation, changes in volume alter the distance between the electrodes and the pressure between the electrodes and skin [40]. Consequently, the capacitance was calculated. The dielectric constant of the skin layer is known to exhibit stable results in the frequency range of 10–50 kHz because of its lower loss coefficients, as shown in Figure 4b. During the experiment, the measured capacitance values were consistent with the actual respiration rate that was being measured. Moreover, real-time monitoring of respiration was possible on a PC monitor throughout the experiment.

Normal respiration data obtained from the wearable respiration sensor were collected prior to normalization and were used to evaluate the reliability of the sensor measurements. Based on the experimental results, the garment respiration sensor yielded valid data. Besides, the garment design ensures that it does not cause discomfort or hinder the subject’s movements, making it suitable for various applications during daily activities. Although the present study utilized a wired connection for accurate measurements, the development of a portable device could allow for convenient and practical usage. If the device is designed in a wireless form, it can be worn and used for measurement during intense or wide-range movements. For example, through miniaturization of the device, it can be made portable, allowing users to wear it during daily activities for data collection. Additionally, with Bluetooth connectivity, data collection can be conveniently achieved.

### 3.2. Characteristics of Breathing According to Motion

**Figure 10:**
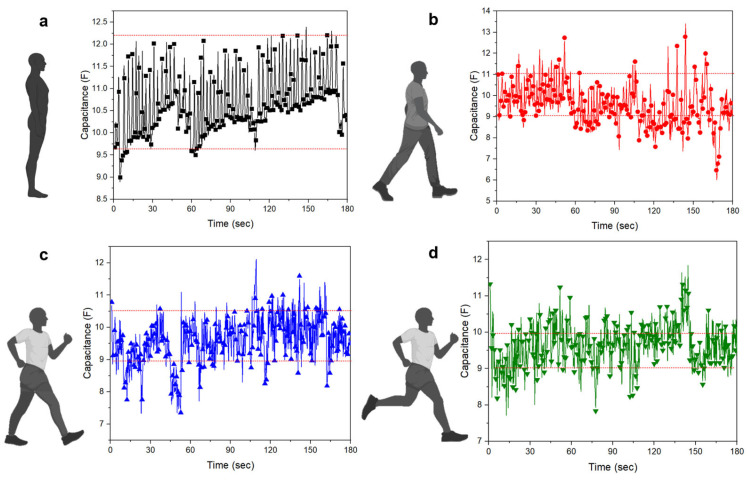
Respiration data according to the four actions: (**a**) standing, (**b**) walking, (**c**) power walking, (**d**) running.

Respiratory measurements were conducted uniformly at the same locations. The participants wore respiratory clothing and completed five sets of 1 min each at the same frequency of 45 kHz. Four actions were proposed: standing, walking, power walking, and running. The data for each movement are shown in Figure 10. The characteristic features of the data were determined by the range of the measurement values. When standing, the range was between 9.75 and 12.25 F, for walking, it was between 9 and 11 F, for power walking, it was between 9 and 10.5 F, and finally, for running, it was between 9 and 10 F with densely packed data. If we convert these values into numerical ranges, the width of standing is 2.5 F, of walking is 2 F, of power walking is 1.5 F, and of running is 1 F. From these data, we can observe that the measurement range becomes narrower as body movements occur. This is because the pressure on the electrodes located on the abdomen and skin decreases as the respiration rate increases, leading to a decrease in the dielectric constant [40]. 

**Figure 11:**
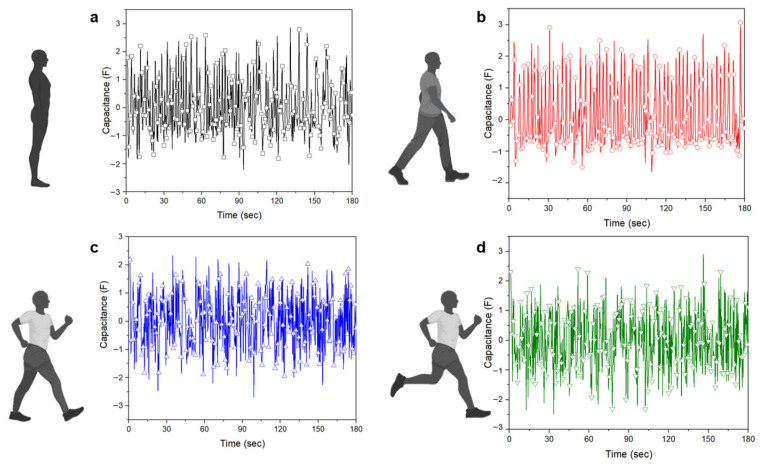
Normalized respiration data according to the four actions: (**a**) standing, (**b**) walking, (**c**) power walking, (**d**) running.

The breathing data characteristics for each action were successfully classified based on the original data. However, in deep learning analysis, it is more effective to normalize the data to a range of 0–1, rather than focusing on each column for training. Therefore, a normalization formula (Equation (2)) was applied to the data to evenly train them (Figure 11).

### 3.3. Classification of the Four Types of Respiration

**Figure 12:**
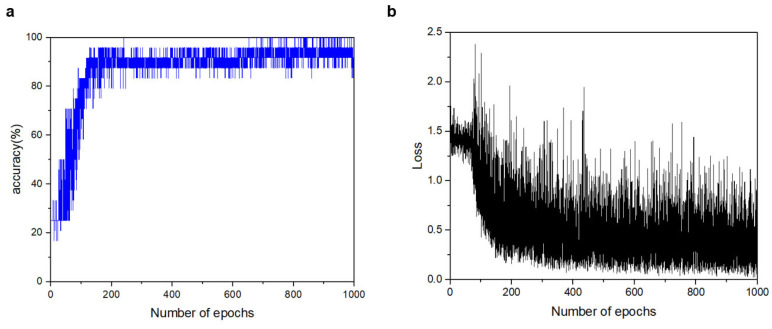
Summary of the 1D CNN classifier: (**a**) validation accuracy, (**b**) training loss.

**Figure 13:**
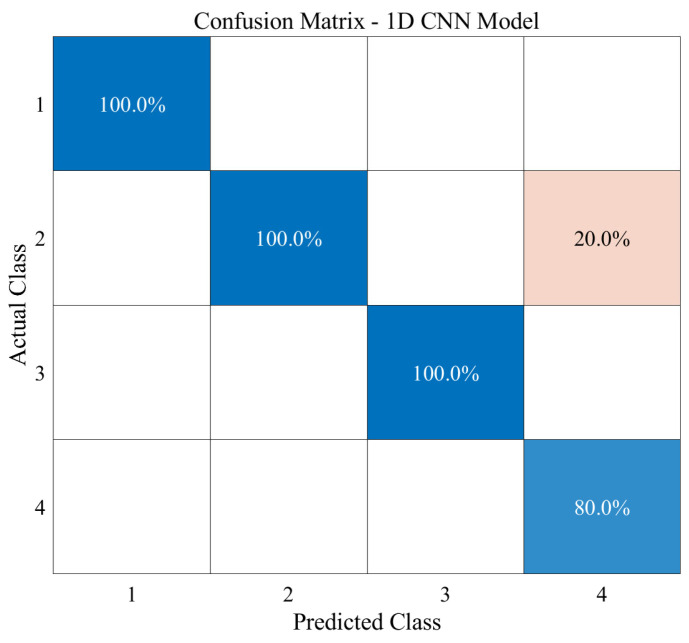
Test accuracy of the 1D CNN classification.

Using a 1D CNN, we trained and classified the entire breathing data for the four normalized actions, totaling 157 sets with 20 samples each: 110 sets for the training set, 24 for the validation set, and 23 new sets for the final test set. By inputting one breathing pattern, we trained the model to classify it into one of four actions. The minibatch size was set to six, and 1000 epochs were conducted. Figure 12 shows the validation accuracy (a) and training loss (b) of models trained using normalized data. The validation and test accuracies were 91.6% and 95.65%, respectively. As the test accuracy was higher than the validation accuracy, we concluded that the model was correctly trained without overfitting. Figure 13 shows the resulting confusion matrix created using the test data. The numbers one to four represent standing, walking, power walking, and running, respectively. Upon examining the matrix, actions one to three were predicted with 100% accuracy, whereas action four was only predicted with 80% accuracy. Therefore, it can be concluded that action four is the most difficult to classify among the four actions in this study.

The reason why classifying running respiration was challenging was due to the confusion with action two, walking. Both walking and running have relatively large strides, leading to similar movements and respiratory patterns. Additionally, in this study, the running performed was more of light jogging rather than sprinting. Therefore, in future research, by analyzing respiratory patterns based on stride, it is expected that more accurate classification of running can be achieved.

## 4. Discussion

This study analyzed the factors that significantly affect the change in capacitance values using a fiber-type capacitive respiration sensor with embroidered electrodes, connectors, and connector connection lines. The conductive yarn used in the embroidery is made of silver-coated nylon filaments, which offer excellent conductivity, low susceptibility to corrosion, and suitability for embroidery [33,34]. Moreover, it is safe for human use without causing harm [16]. The connection lines that link the connectors and electrodes are designed in a zigzag pattern to ensure comfort during garment wear and minimize the impact on movement. Additionally, the electrodes are created using high-density embroidery, providing stability and reliable results during experimentation. 

The distance between the electrodes located in the abdominal position in front of and behind the body, the volume of air in the lungs due to respiration, and the change in the dielectric constant of the skin layer due to the pressure of the electrodes and skin during respiration were examined. The most direct factor was the increase in the dielectric constant of the skin layer due to skin pressure, which resulted in an increase in the capacitance [40]. PED functions as a surrogate for the human lung when worn. Based on the factor loss, PED measurements start at 10 kHz and exhibit a significant increase beyond 50 kHz. Additionally, the dielectric constant of the skin layer introduces noise at higher frequencies, particularly beyond 50 kHz. Therefore, 45 kHz, which falls within the range of 10 kHz to 50 kHz, was chosen as the most suitable frequency for experimentation [16]. Besides, when pressure was applied to a PED with added electrodes and an air layer, the same results were observed for the frequency range with the largest linear variation in the capacitance values (Figure 5b).

To classify the respiratory data based on the four different activities, we designed a 1D CNN that could produce four output classifications with one input. Figure 13 shows that the network can accurately predict the activities of standing, walking, and running, but it incorrectly predicted some of the running data as walking. The difficulty in classifying the respiration of running was due to the potential confusion with walking. Both walking and running involve relatively large strides, which result in similar movements and respiratory patterns. Furthermore, since the data are obtained from actual human subjects rather than machines, there may be limitations in the quantity of measured data. Consequently, to achieve more successful data classification, we used normalized data. By increasing the data in a 1D CNN, the network can overcome these limitations and improve its classification performance. Through training on diverse examples, the network can better capture the variability in the data, leading to improved classification without overfitting. This enables the network to classify new data accurately.

## 5. Conclusions

In this study, we developed a capacitance-based respiratory measurement garment that allows the measurement of four different types of respiration. In addition, we proposed a method for classifying these four types of respiration using a 1D CNN. First, the garment utilizes a capacitance-based approach with textile electrodes, making it lightweight and easy to use; it also provides accurate respiratory data by measuring changes in electrical impedance caused by changes in air pressure and movement of the chest wall. Second, the 1D CNN approach used in this study is not only capable of classifying respiratory data but can also be easily applied to other types of data and is highly scalable to accommodate large datasets. Therefore, this classification system can be applied to various fields beyond respiratory data analysis. With the further development of deep learning techniques and real-time monitoring devices, we anticipate that this system can be utilized with other physiological signals, such as heart rate, to provide a more comprehensive assessment of the body’s functions. In the future, if a device capable of real-time monitoring of respiration data is developed, it is expected to become a completely wearable system that includes bio-signal sensing.

## Figures and Tables

**Table 1:** Specifications of the embroidered capacitance respiratory sensor.

Parameter	Electrodes	Connection Line	Connectors
Dimension (width × height) Density (line/mm)	100 × 50 (mm) 6	25 (mm)	20 × 20 (mm)
Shape of the stitch Length of the stitch (mm) Number of stitches	Running, fill 2, 4 9526	Zigzag 5/1 (width/height)	Running, fill 2, 4 606

## Data Availability

Not applicable.
